# Polydatin inhibits mast cell-mediated allergic inflammation by targeting PI3K/Akt, MAPK, NF-κB and Nrf2/HO-1 pathways

**DOI:** 10.1038/s41598-017-12252-3

**Published:** 2017-09-19

**Authors:** Jing Ye, Hongmei Piao, Jingzhi Jiang, Guangyu Jin, Mingyu Zheng, Jinshi Yang, Xiang Jin, Tianyi Sun, Yun Ho Choi, Liangchang Li, Guanghai Yan

**Affiliations:** 1 Department of Anatomy and Histology and Embryology, Yanbian University Medical College, Yanji, 133002 P.R. China; 2 Department of Respiratory Medicine, Yanbian University Hospital, Yanji, P.R. China; 3 College of Pharmacy, Yanbian University, Yanji, 133002 P.R. China; 4 Department of Anatomy, Medical School, Institute for Medical Sciences, Chonbuk National University, Jeonju, Jeonbuk 561-756 Republic of Korea

## Abstract

Polydatin(PD) shows anti-allergic inflammatory effect, and this study investigated its underlying mechanisms in *in vitro* and *in vivo* models. IgE-mediated passive cutaneous anaphylaxis (PCA) and passive systemic anaphylaxis (PSA) models were used to confirm PD effect *in vivo*. Various signaling pathway proteins in mast cell were examined. RT-PCR, ELISA and western blotting were applied when appropriate. Activity of Lyn and Fyn kinases *in vitro* was measured using the Kinase Enzyme System. PD dose-dependently reduced the pigmentation of Evans blue in the PCA model and decreased the concentration of serum histamine in PSA model, and attenuated the degranulation of mast cells without generating cytotoxicity. PD decreased pro-inflammatory cytokine expression (TNF-α, IL-4, IL-1β, and IL-8). PD directly inhibited activity of Lyn and Syk kinases and down-regulated downstream signaling pathway including MAPK, PI3K/AKT and NF-kB. In addition, PD also targets Nrf2/HO-1 pathway to inhibit mast cell-derived allergic inflammatory reactions. In conclusion, the study demonstrates that PD is a possible therapeutic candidate for allergic inflammatory diseases. It directly inhibited activity of Lyn and Syk kinases and down-regulates the signaling pathway of MAPK, PI3K/AKT and NF-κB, and up-regulates the signaling pathway of Nrf2/HO-1 to inhibit the degranulation of mast cells.

## Introduction

About 20% of the global population is affected by increasing allergic diseases, including atopic dermatitis, allergic rhinitis, allergic asthma, anaphylaxis, and food allergy^1^. Even though the symptoms of these allergic diseases are different, they share similar cellular mechanisms. Mast cells are well known to play a key role in allergic diseases through the production and secretion of allergic mediators, such as histamine, chemokines, cytokines, and growth factors^2^. They are widely distributed throughout vascularized tissues, particularly near surfaces exposed to the external environment, including the skin, airways, conjunctiva and gastrointestinal tract^3–6^. Antigen cross-linking of IgE bound to FcεRI is necessary to trigger the activation of mast cells. B cells stimulated by antigen-presenting cells produce antigen-specific IgE, which binds to FcεRI on mast cell surface^7^. In mast cells, degranulation, production and release of allergic mediators such as histamine, pro-inflammatory cytokines, and various enzymes are achieved through cross-linking of their high affinity surface receptors for IgE^8^. Activation of Src family kinases (Lyn and Syk) causes phosphorylation of phosphoinositide 3-kinase (PI3K), which stimulates Akt and phospholipase C (PLC)γ, followed by calcium mobilization and activation of protein kinase C, mitogen-activated protein kinases (MAPKs) and nuclear factor (NF)-κB^9^.

Histamine is a major factor in acute allergic responses that causes vasodilation and increased vascular permeability, and consequently, leading to edema, development of hypothermia and recruitment of leukocytes. On the late phase responses of allergic inflammation, tumor necrosis factor (TNF)-α, interleukin (IL)-4, IL-1β and IL-8, as chemotactic and pro-inflammatory mediators, are released by mast cells^10^. Terminal microenvironment is changed by these cytokines released from mast cells. Therefore, it is important to reduce these pro-inflammatory cytokines to relive allergic inflammatory symptoms.

MAPKs are highly conserved mediators of eukaryotic signal transducing enzymes that respond to extracellular stimuli and regulate diverse cellular activities in the nucleus. This enzyme family includes extracellular signal-regulated kinase (ERK), c-Jun N-terminal kinases (JNK) and p38 proteins. In mast cells, MAPKs play a crucial role in the regulation of pro-inflammatory cytokines^10^. NF-κB is a transcriptional factor that regulates expression of pro-inflammatory cytokines. For this reason, NF-κB has become a target of anti-inflammatory treatment^11^. Recently, Heme oxygenase-1 (HO-1) has anti-inflammatory properties associated with cytoprotective responses, which are shown to be activated by various phytochemicals involved in beneficial immune responses^12^. Nuclear factor-erythroid 2-related factor 2 (Nrf2) plays major roles in the etiopathogenesis of many cancers and inflammation-related diseases such as inflammatory bowel disease and Parkinson’s disease^13,14^. Transcription factor Nrf2 is a member of the basic leucine zipper NF-E2 family and plays a necessary role in antioxidant response element (ARE)-mediated expression of phase 2 detoxifying enzymes and stress-inducible genes that are indispensable to cellular defense against many chemical insults of endogenous and exogenous origins^15^. In the nucleus, Nrf2 is critical for HO-1 induction and activated by diverse oxidants, pro-oxidants, antioxidants and chemopreventive agents^15^. PI3K, a classical upstream kinase in mTOR pathway, has been implicated in various immune responses and inflammatory processes. The kinase Akt is the main (but not exclusive) intermediate between PI3K and mTOR kinase^16^. Signaling pathways mediated by PI3K/Akt, MAPK, and transcription factors such as activator protein-1 (AP-1), NF-κB, and Nrf2 are the predominant cascades that participate in HO-1 expression. A recent study indicates that HO-1 expression via PI3K/Akt and MAPK signaling pathway induces anti-inflammatory response^17^.

Polydatin (3, 4′, 5-trihydroxystibene-3-β-mono-D-glucoside; PD), also known as polygonin, is a natural component extracted from perennial herb Polygonum cuspidatum and a traditional Chinese herbal medicine. PD is one of the major stilbenoid glucosides in grape juice and red wine. Previously, pharmacological studies and clinical practice show that PD has multiple biological activities and pharmacological effects^18,19^. Previous studies have demonstrated that PD inhibits IgE-mediated passive cutaneous anaphylaxis in mice by stabilizing mast cells through modulating Ca^2+^ mobilization^20^, and attenuates food allergy via store-operated calcium channels in mast cell^18^. PD modulates inflammation by decreasing NF-κB activation and oxidative stress^21^. All results suggest that PD has therapeutic effects on allergic diseases.

In the present study, we aim to evaluate the inhibitory effect of PD on allergic inflammation using *in vitro* and *in vivo* models for immediate-type hypersensitivity. In addition, anti-allergic effects related to the inhibition of mast cell degranulation and inflammatory cytokine expression are investigated using mast cells.

## Results

### PD decreases anti-DNP IgE-mediated PCA and PSA response

**Figure 1:**
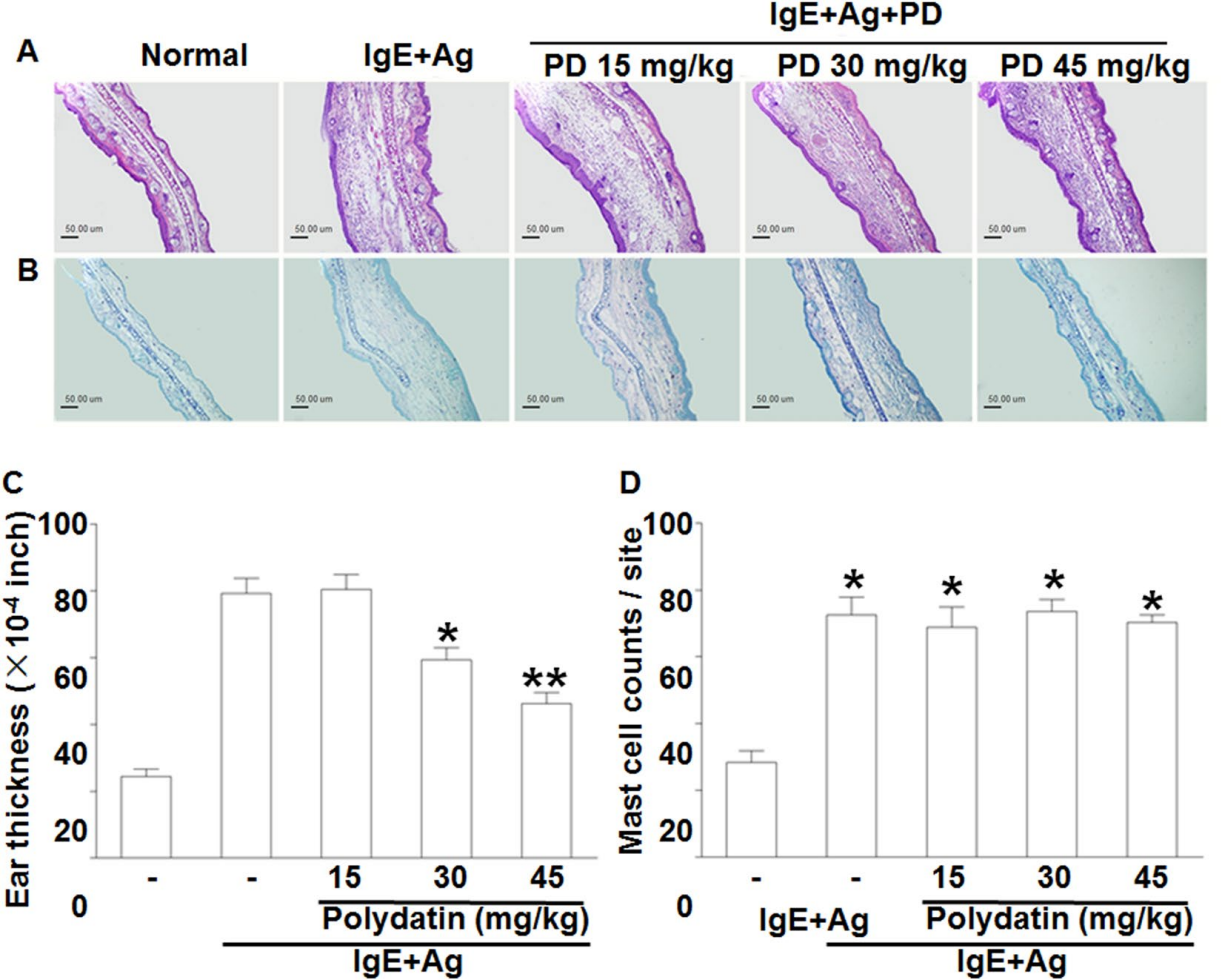
Effect of polydatin on anti-DNP IgE-mediated ear swelling response in mice. The skin on the ears of the mice were sensitized with an intradermal injection of anti-DNP IgE. Polydatin was administered intraperitoneally at doses of 15, 30 and 45 mg/kg BW 1 h before intravenous injection of DNP-HSA. Thirty minutes later, the ears were harvested for histology. (**A**,**B**) Representative photomicrographs of ear sections stained with hematoxylin & eosin and toluidine blue (magnification, ×200). Normal, normal mice; Control, sensitization with anti-DNP IgE and challenge with DNP-HAS mice; Polydatin was administered intraperitoneally at doses of 15, 30 and 45 mg/kg BW 1 h before challenge with DNP-HSA. (**C**) Ear thickness measured with a dial thickness gauge. (**D**) The number of mast cells counted at dermis. The data were expressed as means ± S.E.M. of five independent experiments. *p < 0.05 and **p < 0.01 compared with the group sensitized with anti-DNP IgE and challenged with DNP-HAS in the absence of polydatin.

To study immediate-type allergic reactions, PCA animal model was used. After challenges of antigen, a blue spot was developed at the sensitized site because of increased vascular permeability caused by histamine released from mast cells. PD (15–40 mg/kg) inhibited dye extravasation in a dose-dependent manner (Table 1). Similarly, PD inhibited the concentration of histamine in anti-DNP IgE-mediated PSA mice (Table 2). Ear thickness was increased by injection of antigen and decreased by PD (Fig. 1A,C). To confirm whether the reduction of vascular permeability was caused by the damage of mast cells, ear sections were stained with toluidine blue (Fig. 1B). The number of mast cells at PCA site was increased by injection of antigen but not decreased by PD (Fig. 1D). These results suggest that PD inhibits activation of mast cells instead of the number of mast cells to decrease anti-DNP IgE-mediated PCA and PSA response.

**Table 1:** Effect of polydatin on anti-DNP IgE-mediated passive cutaneous anaphylaxis in mice

Treatment (mg/kg BW)		Anti-DNP IgE	Amount of Evans blue (μg/g)	Inhibition (%)
Normal		−	45.68 ± 3.29	—
Polydatin	15	−	48.34 ± 4.02	—
	30	−	46.37 ± 5.12	—
	45	−	49.59 ± 6.34	—
Control		+	234.57 ± 11.86	—
Polydatin	15	+	213.46 ± 10.38	12.58
	30	+	162.45 ± 9.28^*^	38.55
	45	+	145.23 ± 9.12^**^	49.37

Note: Amount of Evans blue was presented as means ± S.E.M. of five independent experiments. Inhibition rate (%) = {1-(T-B)/(C-N)} ×100. Control (C): IgE (+), polydatin (−); Normal (N): IgE (−), polydatin (−); Test (T): IgE (+), polydatin (+); Blank (B): IgE (−), polydatin (+). *p < 0.05 and **p < 0.01 com*p*ared with the group sensitized with anti-DNP IgE and challenged with DNP-HAS in the absence of polydatin.

**Table 2:** Effect of polydatin on anti-DNP IgE-mediated passive systemic anaphylaxis in mice.

Treatment (mg/kg BW)		Anti-DNP IgE	Histamine concentration (μg/ml)	Inhibition (%)
Normal		−	0.10 ± 0.02	—
Polydatin	15	−	0.09 ± 0.01	—
	30	−	0.12 ± 0.03	—
	45	−	0.11 ± 0.02	—
Control		+	1.32 ± 0.16	—
Polydatin	15	+	1.13 ± 0.21	14.75
	30	+	0.83 ± 0.19^*^	39.34
	45	+	0.61 ± 0.14^**^	59.02

Note: Serum histamine concentration was presented as means ± S.E.M. of five independent experiments. Inhibition rate (%) = {1-(T-B)/(C-N)} ×100. Control (C): IgE (+), polydatin (−); Normal (N): IgE (−), polydatin (−); Test (T): IgE (+), polydatin (+); Blank (B): IgE (−), polydatin (+). *p < 0.05 and **p < 0.01 com*p*ared with the group sensitized with anti-DNP IgE and challenged with DNP-HAS in the absence of polydatin.

### PD decreases degranulation, histamine release and intracellular calcium level of mast cells

**Figure 2:**
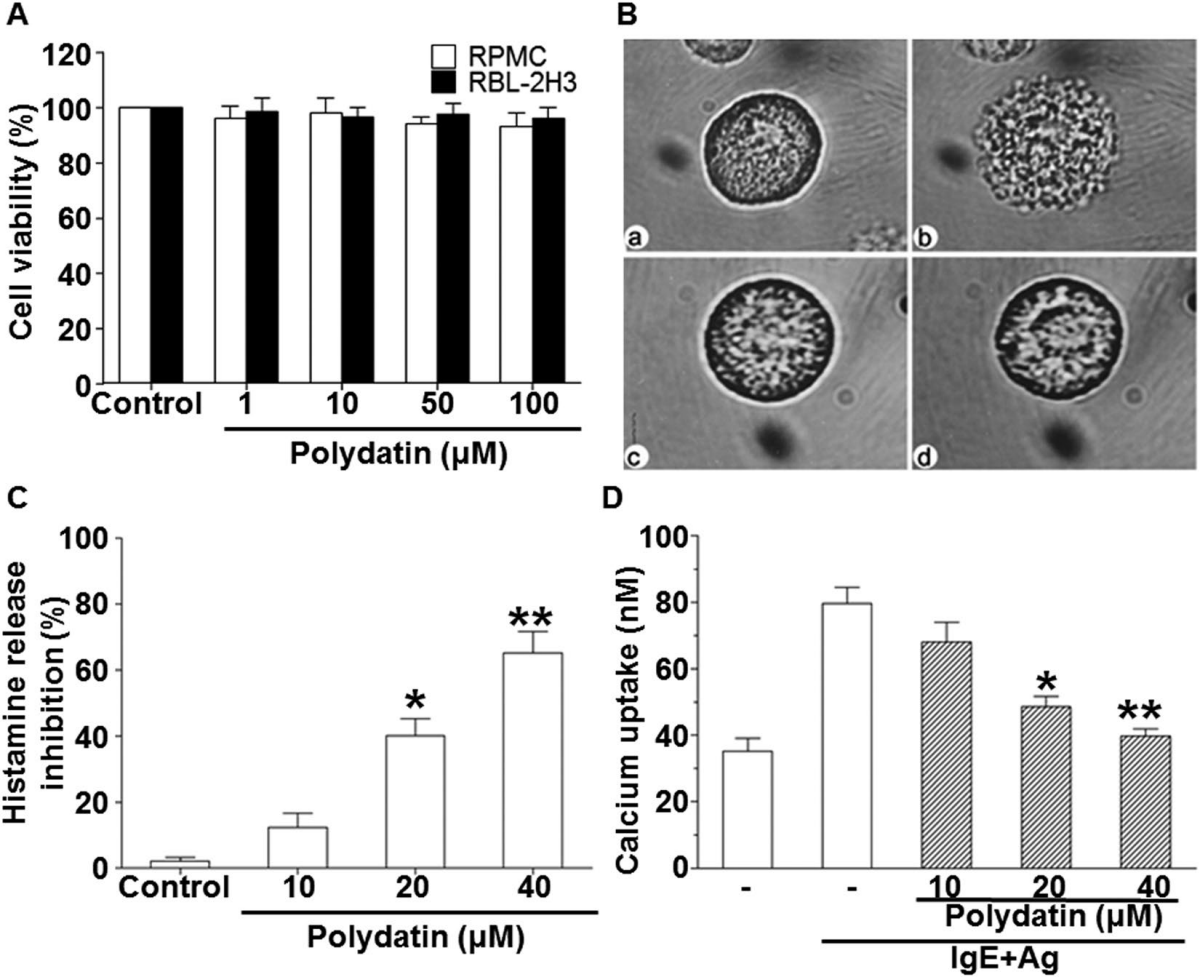
Effect of polydatin on cell viability and degranulation of rat peritoneal mast cells (RPMC and RBL-2H3 cells). (**A**) Cell viability represented by relative absorbance compared to control. RBL-2H3 and purified RPMC cells (2 × 10^4^ cell/well in 96-well plates) were treated with various concentrations of polydatin. (**B**) Effect of polydatin on anti-DNP IgE-mediated degranulation of RPMCs (Magnification, ×1,000). (a) Normal RPMCs in HEPES-Tyrode buffer; (b) RPMC were sensitized with 10 μg/ml anti-DNP IgE for 6 h and challenged with 100 ng /mL DNP-HAS; (c) RPMC incubation with 40 μM polydatin at 37 °C for 30 min; (d) prior to the challenge with 100 ng /mL DNP-HAS preincubated with 40 μM polydatin at 37 °C for 30 min. (**C**) Histamine release rate and (**D**) calcium uptake by purified RPMCs sensitized with 10 μg/ml anti-DNP IgE for 6 h and preincubated with polydatin (10, 20 and 40 μM) at 37 °C for 30 min prior to challenge with DNP-HSA (100 ng / mL). Data were presented as means ± S.E.M. of five independent experiments. *p < 0.05 and **p < 0.01 compared with the group stimulated with anti-DNP IgE and challenged with DNP-HSA in the absence of polydatin.

To check the effect of PD on the cell viability, MTT assay was performed. RBL-2H3 and RPMC cells were treated with various concentrations (1–100 μM) of PD and incubated for 24 h. The data showed that up to 100 μM of PD did not alter the viability of RBL-2H3 and RPMC cells (P > 0.05) (Fig. 2A). In addition, PD attenuated anti-DNP IgE-mediated degranulation in RBL-2H3 cells in a dose-dependent manner (Fig. 2B). The release of histamine by RBL-2H3 cells was inhibited by PD in a dose-dependent manner (P < 0.05) (Fig. 2C). To investigate the mechanisms of PD on the reduction of histamine release, we assayed intracellular calcium. The intracellular calcium level in RBL-2H3 cells was rapidly increased after exposure to DNP-HSA, but PD reduced intracellular calcium level (P < 0.05) (Fig. 2D). The results indicate that PD decreases degranulation, histamine release and intracellular calcium level of mast cells.

### PD reduces the expression of inflammatory cytokines and the phosphorylation of signaling proteins

**Figure 3:**
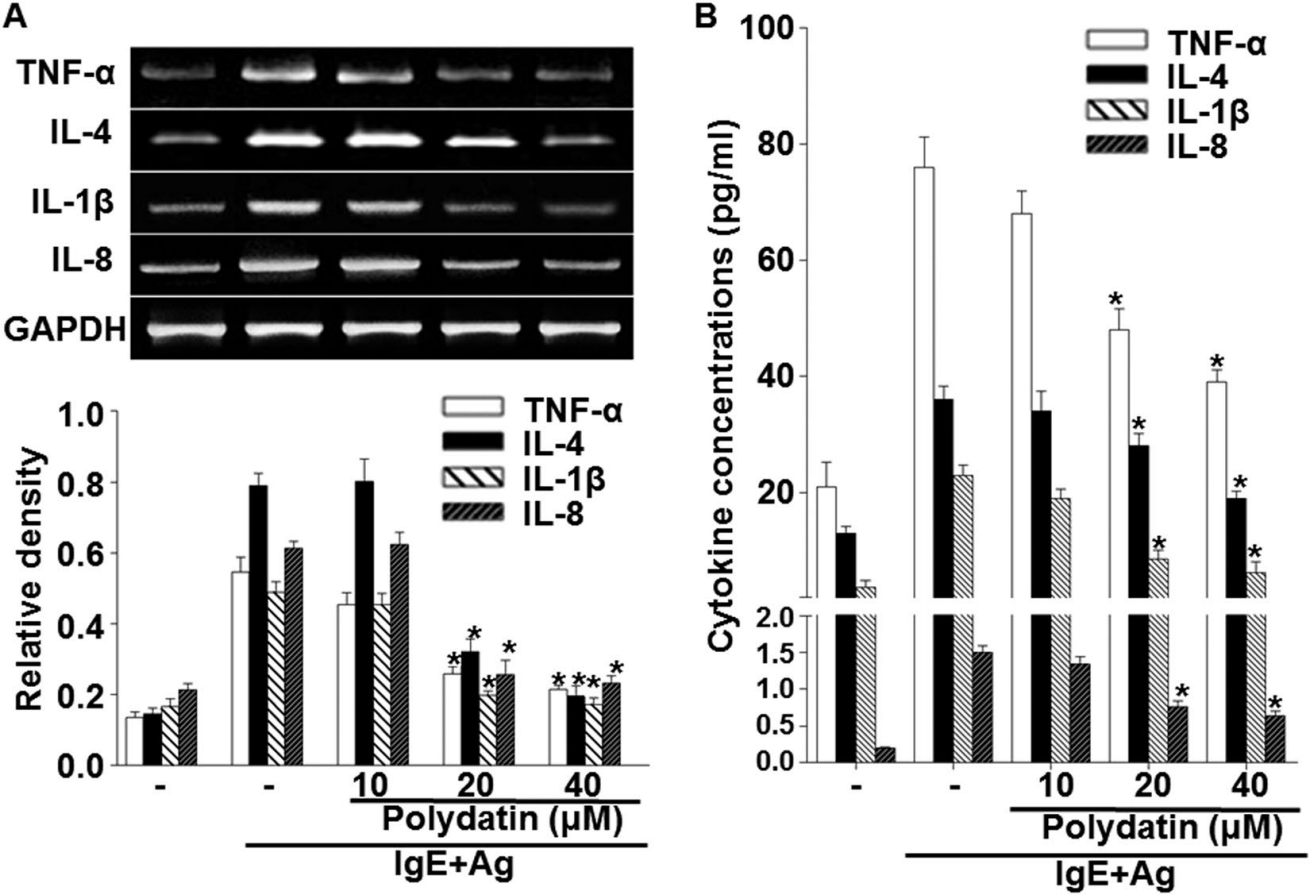
Effect of polydatin on gene and protein expression of pro-inflammatory cytokines (TNF-α, IL-4, IL-1β and IL-8) in RBL-2H3 cells. RBL-2H3 cells were stimulated with 10 μg/ml anti-DNP IgE for 6 h and challenged with 100 ng /mL DNP-HAS in the absence or presence of polydatin (10, 20 and 40 μM). (**A**) Gene expression of these cytokines were measured by RT-PCR. Cropped gels are displayed. (**B**) The level of cytokines in the supernatant measured by ELISA. Data were presented as means ± S.E.M. of five independent experiments. *p < 0.05 compared with the group stimulated with anti-DNP IgE and challenged with DNP-HSA in the absence of polydatin.

**Figure 4:**
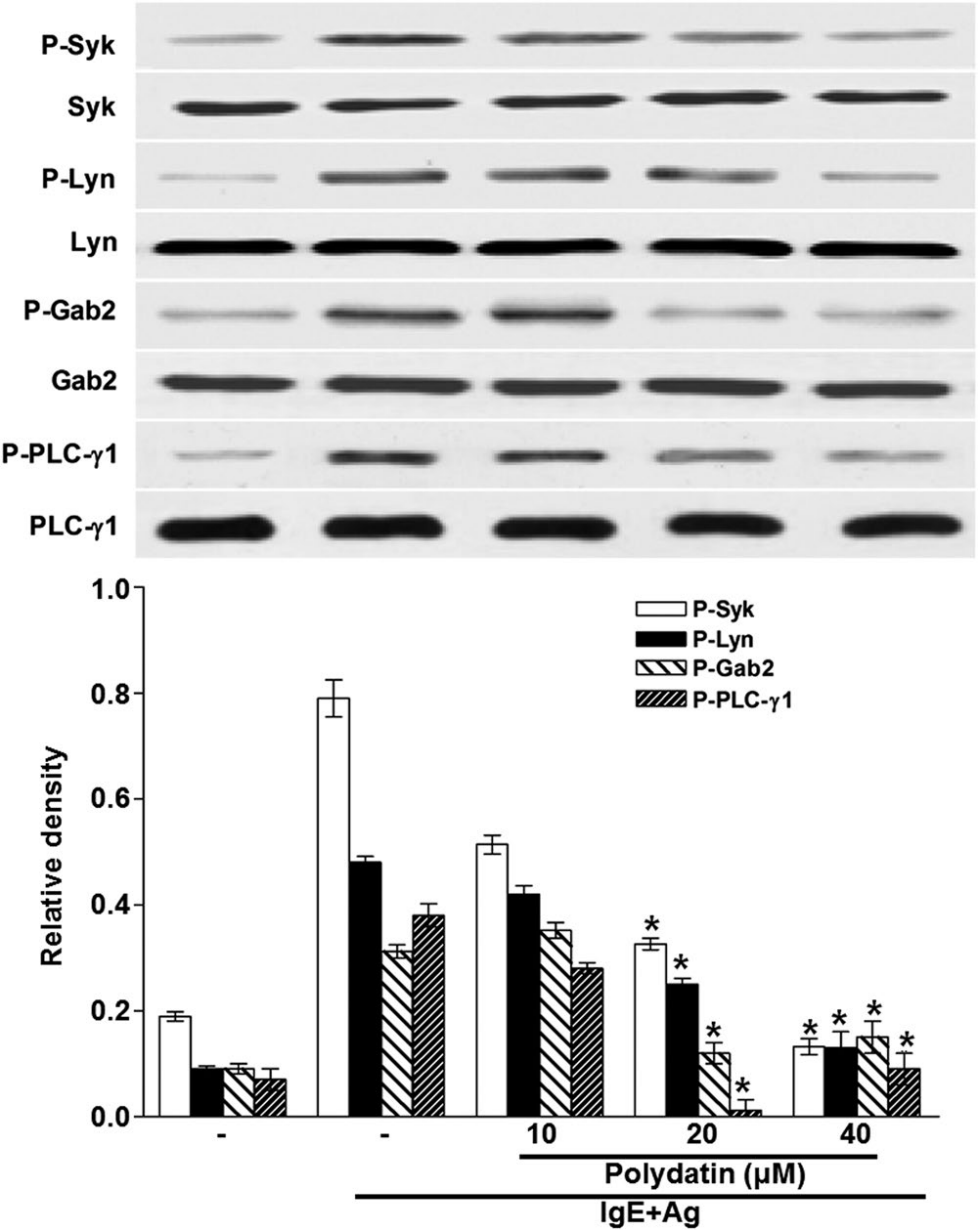
Effect of polydatin in activating the phosphorylation of Syk and Syk-mediated downstream molecules such as Lyn, Gab2 and PLC-γ1 in RBL-2H3 cells. RBL-2H3 cells were stimulated with 10 μg/ml anti-DNP IgE for 6 h and challenged with 100 ng /mL DNP-HAS in the absence or presence of polydatin (10, 20 and 40 μM). Cropped blots are displayed. Data were presented as means ± SEM of five independent experiments. *p < 0.05 compared with the group stimulated with anti-DNP IgE and challenged with DNP-HSA in the absence of polydatin.

**Figure 5:**
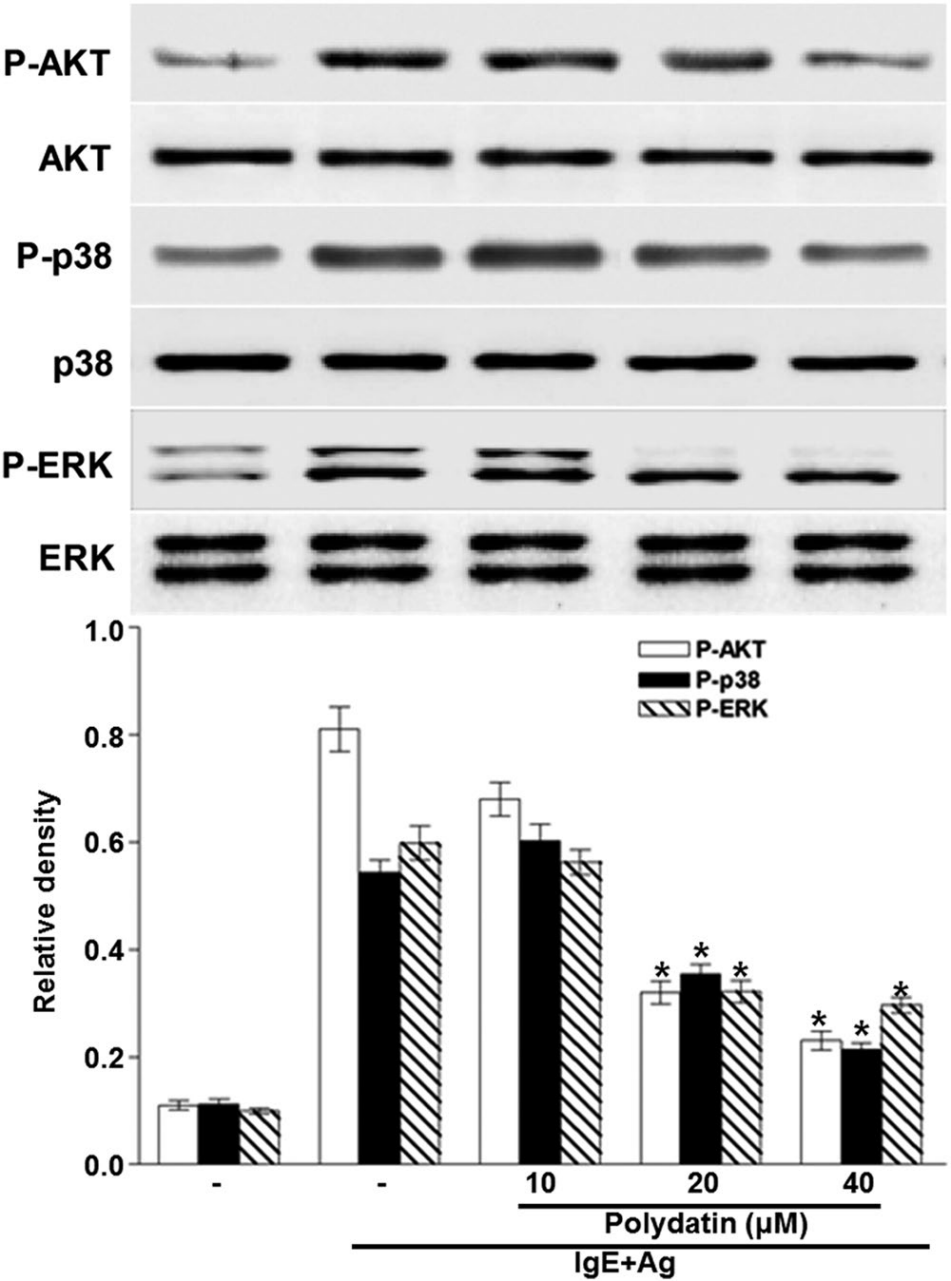
Effect of polydatin on the activation of AKT and MAPKs. RBL-2H3 cells were stimulated with 10 μg/ml anti-DNP IgE for 6 h and challenged with 100 ng /mL DNP-HAS in the absence or presence of polydatin (10, 20 and 40 μM). Cropped blots are displayed. Data were presented as means ± S.E.M. of five independent experiments. *p < 0.05 compared with the group stimulated with anti-DNP IgE and challenged with DNP-HSA in the absence of polydatin.

Mast cell-derived pro-inflammatory cytokines, especially TNF-α, IL-6, IL-1β and IL-8 have a critical biological role for inducing delayed type hypersensitive allergic and inflammatory responses. Therefore, we examined the effects of PD on the expression and release of inflammatory cytokines such as TNF-α, IL-4, IL-1β and IL-8 in RBL-2H3 cells by real-time PCR and ELISA. The mRNA expression and secretion of these cytokines were elevated by activation of FcεRI (P < 0.05), but was suppressed by PD in a dose dependent manner (P < 0.05) (Fig. 3A,B). To detect the phosphorylation of signaling proteins such as Syk, Lyn, Gab2, PLC-γ and AKT, Western blotting was performed. PD reduced the phosphorylation of Lyn, Syk, Gab2, PLC-γ and AKT protein at a dose of 10 μM (Figs 4 and 5). The results suggest that PD reduces the expression of inflammatory cytokines and the phosphorylation of signaling proteins.

### PD inhibits the nuclear translocation of NF-κB, reduces phosphorylation of ERK and p38, and promotes the expression of Nrf2 and HO-1

**Figure 6:**
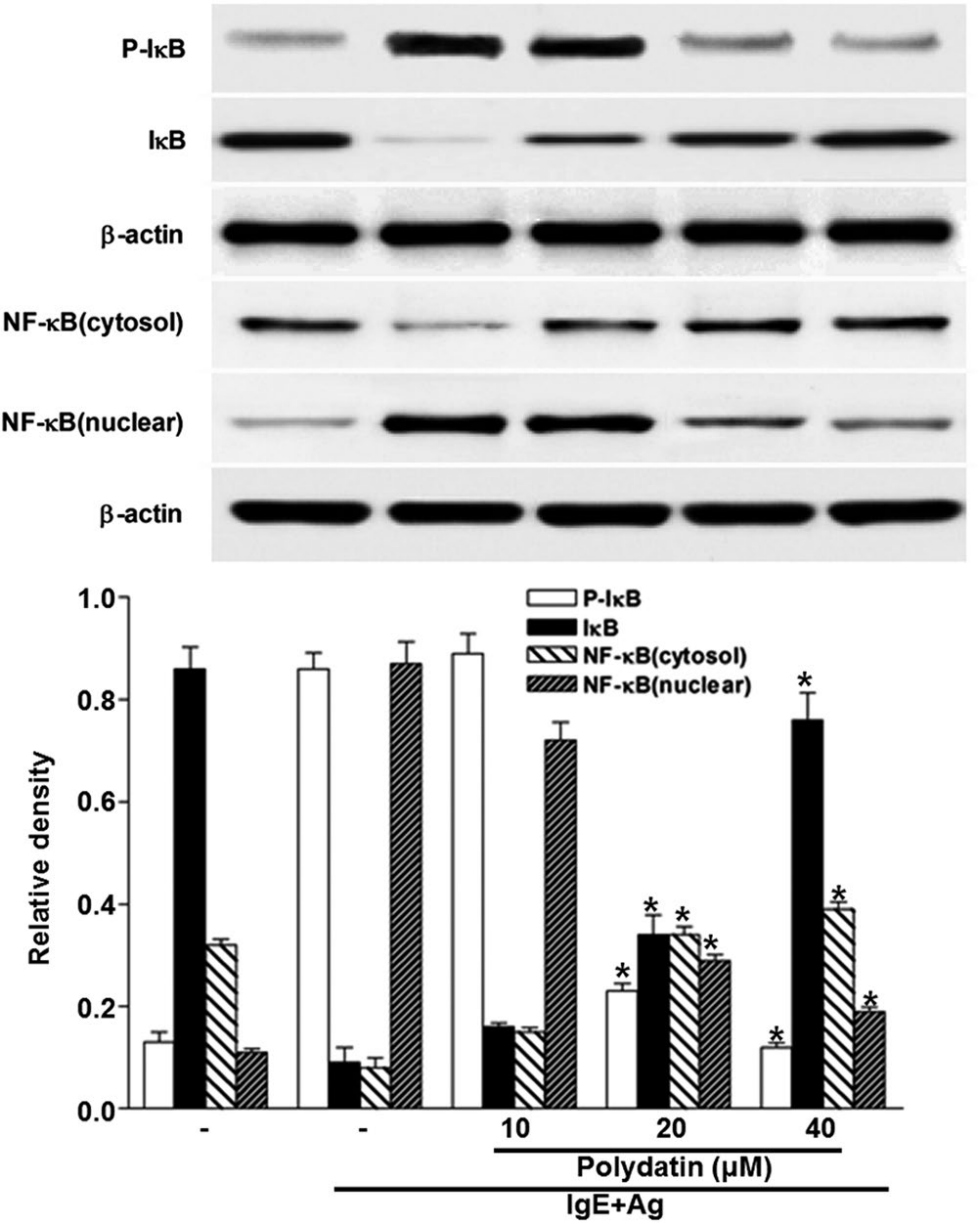
Effect of polydatin on NF-κB activation in RBL-2H3 cells. RBL-2H3 cells were stimulated with 10 μg/ml anti-DNP IgE for 6 h and challenged with 100 ng /mL DNP-HAS in the absence or presence of polydatin (10, 20 and 40 μM). Cropped blots are displayed. Data were presented as means ± S.E.M. of five independent experiments. *p < 0.05 compared with the group stimulated with anti-DNP IgE and challenged with DNP-HSA in the absence of polydatin.

**Figure 7:**
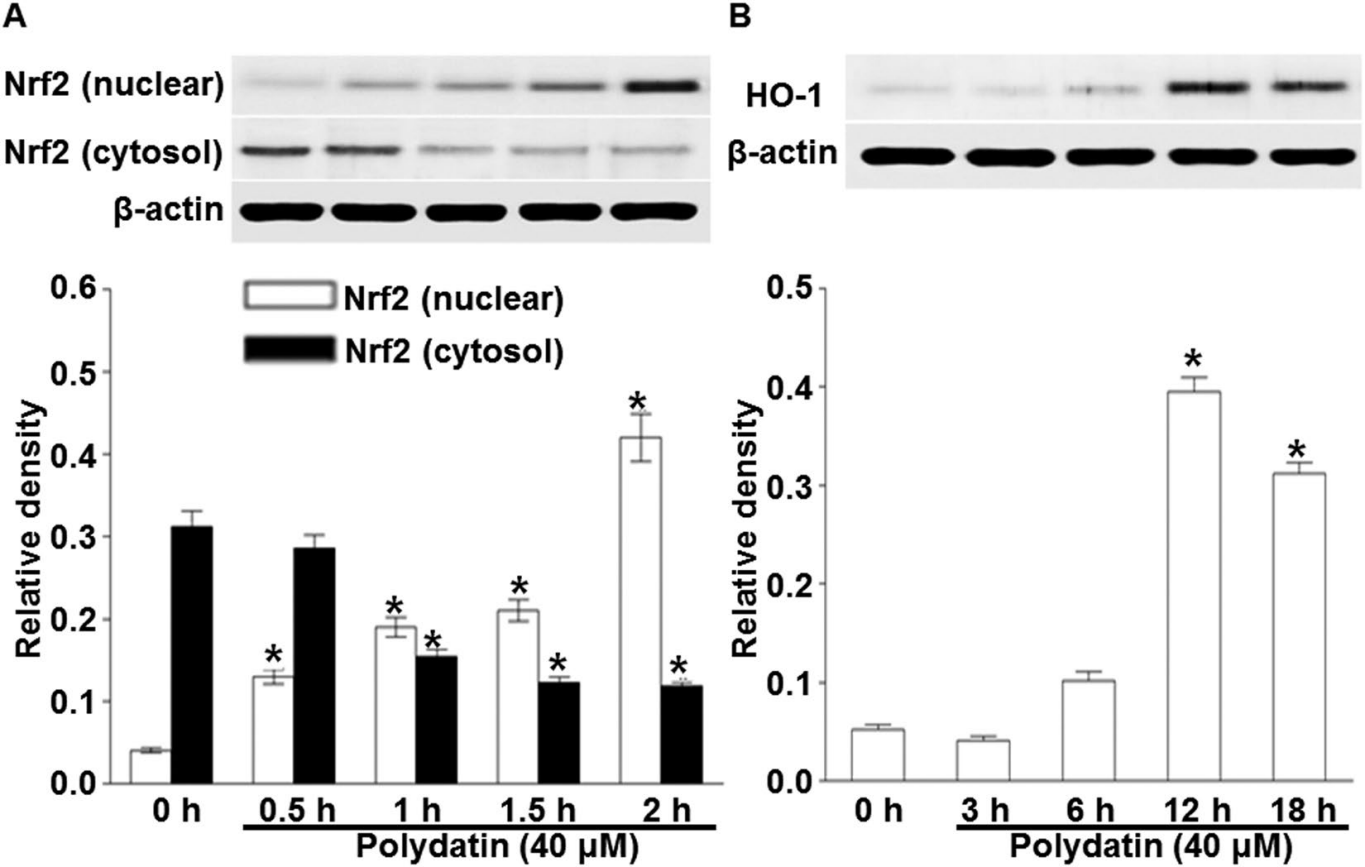
Effect of polydatin on the expression of HO-1 and Nrf2 proteins in RBL-2H3 cells. RBL-2H3 cells were stimulated with 10 μg/ml anti-DNP IgE for 6 h and challenged with 100 ng /mL DNP-HAS in the presence of 40 μM polydatin at the indicated time points (0, 0.5, 1, 1.5, 2 h) for Nrf2 and (0, 3, 6, 12, 18 h) for HO-1. Cropped blots are displayed. Data were presented as means ± S.E.M. of five independent experiments. *p < 0.05 significant compared with the group stimulated with anti-DNP IgE and challenged with DNP-HSA in the presence of polydatin.

To find out the mechanism responsible for the inhibitory effect of PD on cytokine expression, we investigated the effect of PD on NF-κB translocation and MAPK phosphorylation using Western blotting. Stimulation of RBL-2H3 cells with anti-DNP IgE induced translocation of p65 NF-κB to the nucleus following the degradation of IκBα. The nuclear translocation of NF-κB was increased by anti-DNP IgE binding and hindered by PD. To anticipate the target point at which PD inhibited the expression of pro-inflammatory cytokines, we investigated the effect of PD on the activation of representative signaling proteins in mast cells such as IκBα. The data showed that PD hindered activation of NF-κB and IκBα (Fig. 6). Moreover, anti-DNP IgE induced phosphorylation of ERK and p38, and PD reduced phosphorylation of ERK and p38 (Fig. 5). Nrf2-HO-1 pathway mediates the anti-allergic actions of quercetin in rodent mast cells^22^. HO-1 inhibits cytokine production by activated mast cells^23^ and stabilizes mast cells to play an anti-inflammatory role^23^. Previous studies have demonstrated that PD promotes Nrf2-ARE anti-oxidative pathway through activating Sirt1 in rat glomerular messangial cells^24^ and up-regulated the expression and activity of heme oxygenase (HO-)1 in lung tissue of septic mice^25^. All these studies suggest that PD targets Nrf2-HO-1 pathway to play inhibitory roles in inflammatory diseases. In our study, PD also promoted the expression of Nrf2 and HO-1 proteins in mast cells in a time-dependent manner (Fig. 7). These results indicate that PD inhibits the nuclear translocation of NF-κB, reduces phosphorylation of ERK and p38, and promotes the expression of Nrf2 and HO-1.

### PD suppresses activation of Lyn and Syk kinases

**Figure 8:**
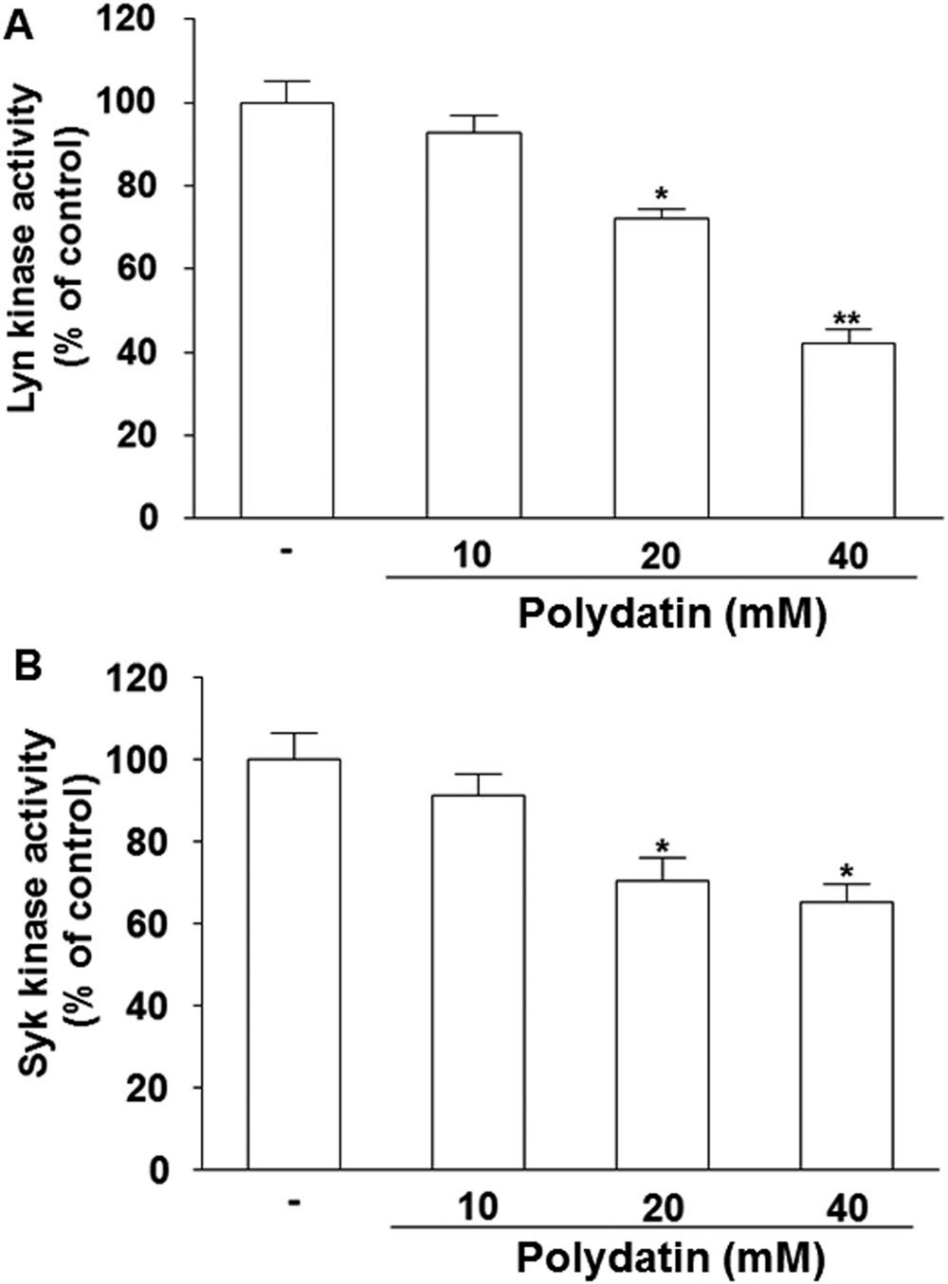
Effect of polydatin on activity of Lyn and Syk in RBL-2H3 cells. The activity of Lyn (**A**) and Syk (**B**) was measured by the ADP-GloTM kinase assay. Data were presented as means ± S.E.M. of five independent experiments. *p < 0.05 and **p < 0.01 compared with the group substrate-alone group in the absence of polydatin.

Ag-mediated aggregation of IgE-occupied FceRI on mast cell surface leads to activation of Src family kinases. This in turn phosphorylates immunoreceptor tyrosine-based activation motifs (ITAMs), leading to activation of Syk kinase in the early receptor-proximal signaling event^26,27^. Therefore, we measured the effect of PD on activity of Src family kinases Lyn and Syk by using an *in vitro* enzyme system. PD significantly inhibited the activation of Lyn and Syk kinases (Fig. 8A,B), indicating that PD can inhibit the activation of Src family kinases.

## Discussion

Natural products have been considered as valuable sources for drug development. There are a lot of reports about the importance of drug discovery from natural medicines. PD is one of the major stilbenoid glucosides in grape juice and red wine^28^. It is a traditional medicinal herb similar to its analog resveratrol in Asia, which has been used for anti-inflammatory, neuroprotective, and lipid-lowering actions^29,30^. Even though intake of natural medicines has been used as one of the most general therapeutic methods, ingestion of potent single compounds is better through increasing biological effects and alleviating side effects. Our study demonstrates the anti-allergic inflammatory effect of PD using *in vitro* and *in vivo* models.

Our results show that PD plays important roles in immediate-type hypersensitivity, and IgE-dependent mast cell activation leads to the secretion of various preformed mediators. In the presence of allergen exposure, mast cells are activated, leading to degranulation and secretion of histamine, cytokines, protease and chemokines by mast cells. Therefore, mast cells are key targets for the development of medicines for allergic disorders^31^. Histamine is one of the mediators for acute inflammatory and immediate hypersensitivity responses to cause allergic symptoms such as edema warmth and erythema^32^. In the present study, we use RBL-2H3 and RPMC cells to explore the anti-allergic inflammatory effects of PD on mast cells. We use histamine assay on RPMC cells to measure mast cell degranulation. As a feasible therapeutic strategy to weaken mast cell degranulation for the treatment of allergic disorders, PD inhibits mast cell degranulation in a dose-dependent manner without causing cytotoxic effects. Anti-DNP IgE-induced IgE-mediated PCA is positively associated with histamine release from mast cells and suitable animal models for immediate-type hypersensitivity. In the animal model, ear sections are stained with hematoxylin & eosin or toluidine blue, and ear thickness is swelling. These symptoms are reduced by PD. From these results, we infer that PD suppresses the immediate-type allergic reaction through the inhibition of mast cell degranulation, especially the release of histamine.

Correlations between degranulation and intracellular calcium levels in mast cells demonstrate the regulatory role of various calcium-dependent proteins^33^. Regulation of intracellular calcium is critical to histamine release by mast cells. Calcium movements across membranes of mast cells represent a major target for effective anti-allergic drugs, as these are essential events linking stimulation to secretion. Calcium is a crucial secondary messenger in mast cell signaling^34^. Intracellular calcium level regulates exocytosis from mast cells and expression of inflammatory cytokines. In the present study, we have evaluated the effects of PD on DNP-HSA-induced histamine release and intracellular calcium. Mode of action of PD is possibly related with the prevention of histamine release from mast cells due to inhibition of intracellular calcium. Our results show an attenuation of intracellular calcium in mast cells following PD treatment, being consistent with other reports^35^. In the present study, intracellular calcium level in RPMC cells stimulated with DNP-IgE is rapidly elevated, while PD blocks calcium influx. According to these observations, we speculate that decreased intracellular calcium might be involved in the inhibitory effect of PD on histamine release.

Mast cell-derived pro-inflammatory cytokines, especially TNF-α, IL-4, IL-1β and IL-8 have critical biological roles in allergic inflammation^36^. Mast cells are one of the major sources of TNF-α in human dermis. They are necessary in the progression to chronic allergic inflammation^37^. Expression of pro-inflammatory cytokines was increased through antigen-IgE cross-linking. TNF-α contained in the granules of mast cells stimulates adaptive immunity by activating NF-κB and transendothelial migration of immune cells^38^. NF-κB is a key transcription factor that stimulates the expression of pro-inflammatory cytokines^39^. Our results show that degradation of IκBα and translocation of NF-κB into the nucleus are stimulated by antigen-IgE cross-linking. This process is attenuated by PD. As a result, PD hinders the expression of pro-inflammatory cytokines by blocking the degradation of IκB and translocation of NF-κB. TNF-α promotes inflammation, granuloma formation and tissue fibrosis and is thought to be an initiator of cytokine-related inflammatory states by stimulating cytokine production^40^. IL-4, a major Th2 cytokine that is necessary for allergic responses, drives the generation of IgE in plasma B cells^41^. IL-1β aggravates auto-inflammatory and allergic diseases such as contact hypersensitivity, atopic dermatitis, and bronchial asthma^42^. Our results show that PD suppresses the expression of pro-inflammatory cytokines by blocking NF-κB.

Signaling pathways of mast cells have been extensively studied^43^. We report in this study that IgE sensitization induces degranulation in mast cells through the activation of signaling pathways mediated by NF-κB, MAPK, and Akt. IgE-induced degranulation in mast cells is associated with activation of FcεRI receptor, and this activation induces the release of various inflammatory mediators such as TNF-α and leukotrienes via phosphorylation of Lyn / Syk pathway^44^. In turn, activation of Syk increases intracellular Ca^2+^ and induces the activation of MAPK family. PLCγ hydrolyzes phosphatidylinositol 4,5-bisphosphate (PIP2) to diacylglycerol (DAG) and IP3. DAG is reported to activate PKC, which stimulates IKK complex, and IP3 ultimately triggers extracellular calcium influx^45^. IKK stimulation and increased intracellular calcium level provoke the secretion and expression of allergic mediators. Akt pathway also activates IKK complex. Lyn and Syk are important intracellular mediators in early signaling after FcεRI receptor activation. In the present study, Lyn and Syk are markedly inhibited by PD, supporting the notion that it is a primary target of PD. In support of this observation, PD significantly reduces the phosphorylation of ERK and p38, which are downstream effectors of FcεRI. Activation of NF-κB requires phosphorylation and proteolytic degradation of inhibitory protein IκBα, an endogenous inhibitor that binds to NF-κB in the cytoplasm. To evaluate the mechanisms of inhibition of PD on expression of pro-inflammatory cytokines, we have examined the effect of PD on NF-κB pathway. In the present study, PD decreases the degradation of phosphorylated IκBα and nuclear translocation of NF-κB. These results indicate that inhibitory effects of PD on inflammatory cytokines are due to the regulation of NF-κB pathway. MAPKs represent an important point of convergence for multiple signaling pathways that are activated in inflammation, immunity, cell death and proliferation^46^. MAPK family is fundamental in regulating multiple cell functions such as cytokine expression, proliferation, and apoptosis. Although ERK and p38 MAPK are shown to mediate IgE-induced pro-inflammatory gene expression in mast cells recently, Akt is observed to be activated in response to IgE for the first time in mast cells^47^. The expression of pro-inflammatory cytokines is known to be regulated by MAPKs in mast cells. A number of studies have shown that multiple phosphorylation cascades participate in the regulation of the translocation of Nrf2 and Nrf2-mediated HO-1 gene expression^48^. The reports by Yan *et al*.^49^ and Dai *et al*.^50^ show that inflammation could be affected through Nrf2/HO-1 pathways. HO-1 is an inducible enzyme that catalyzes the rate-limiting step in the oxidative degradation of cellular heme into carbon monoxide, biliverdin and free iron. It provides a host defense mechanism that protects against oxidative stress and contributes to the anti-inflammatory activity. In addition, it is reported that Nrf2 signaling pathways negatively regulate NF-κB-mediated inflammatory responses^51^. In the present study, PD causes overexpression of HO-1 with the elevation of Nrf-2 translocation to the nucleus in mast cells. These results indicate that PD attenuates inflammatory responses by enhancing antioxidant status induced by Nrf-2 activation.

In conclusion, the present study demonstrates that PD decreases degranulation of mast cells in a dose-dependent manner through attenuation of intracellular calcium level. PD also suppresses anaphylactic symptoms in PCA model.

PD directly inhibits activity of Lyn and Syk kinases and down-regulates downstream signaling pathway including MAPK, PI3K/AKT and NF-kB to inhibit mast cell-derived allergic inflammatory reactions. We speculate that anti-allergic inflammatory effects of PD are due to the blockade of IKK complex. In addition, we assume that PD also targets Nrf2/HO-1 pathway to play inhibitory role in mast cells. Overall, PD is a potential therapeutic candidate for allergic disorders through the inhibition of degranulation and pro-inflammatory cytokine expression in mast cells.

## Materials and Methods

### Animals

Specific pathogen-free (SPF) 7-week-old inbred male BALB/c mice were purchased from House Section of Yanbian University Health Science Center (Yanji, China). The mice were maintained in an animal facility under standard laboratory conditions for 1 week prior to experiments, and provided with water and standard chow ad libitum. Throughout the study, the animals were housed 3–5 per cage in a laminar air-flow cabinet maintained under temperature of 22 ± 2 °C and relative humidity of 55 ± 5%. The experiments were performed in compliance with the guidelines approved by Institutional Animal Care and Use Committee of Yanbian University. All methods were performed in accordance with the relevant guidelines and regulations.

### Reagents and cell culture

PD (purity, 98%) was obtained from TW Reagent Co., Ltd (Shanghai, China). Dulbecco’s Modified Eagle’s Medium (DMEM) and fetal bovine serum (FBS) were purchased from Invitrogen (Thermo Fisher Scientific, Waltham, MA, USA). 3- (4,5-Dimethyl-2-thiazolyl) -2,5-diphenyl-2H-tetrazolium bromide (MTT) and triton X-100 were purchased from Sigma-Aldrich (St. Louis, MO, USA). All other reagents were of analytical reagent grade. Anti-dinitrophenyl (DNP) IgE, DNP human serum albumin (HSA), Percoll solution was purchased from Pharmacia (Uppsala, Sweden). Dulbecco’s Modified Eagle’s Medium (DMEM) and fetal bovine serum (FBS) were purchased from Invitrogen (Thermo Fisher Scientific, Waltham, MA, USA). RBL-2H3 cells and rat peritoneal mast cells (RPMCs) were grown at 37 °C in 5% CO_2_ in DMEM supplemented with 10% heat-inactivated FBS, 100 U/ml penicillin G, 100 μg/ml streptomycin.

### Preparation of RPMCs

To isolate RPMCs, rats were anesthetized with ether and injected with 10 mL of calcium-free N-(2-hydro-xyethyl) piperazine-N-2-ethanesulfonic acid (HEPES)-Tyrode buffer (137 mM NaCl, 5.6 mM glucose, 12 mM NaHCO_3_, 2.7 mM KCl, 0.3 mM NaH_2_PO_4_ and 0.1% gelatin) into the peritoneal cavity, and the abdomen was gently massaged for approximately 90 s. The peritoneal cavity was opened carefully, and the fluid containing peritoneal cells was collected by a Pasteur pipette, and RPMCs were purified by using a Percoll (Pharmacia, Uppsala, Sweden) as described previously^52^. RPMC preparations were about 95% pure as assessed by toluidine blue staining and more than 98% of the cells were viable as judged by trypan blue uptake. Purified mast cells (1 × 10^6^ cells/ml) were resuspended in HEPES-Tyrode buffer.

### Cell viability

Mast cell viability was determined by colorimetric analysis based on the conversion of MTT. Water-soluble MTT is converted into water-insoluble formazan by mitochondrial dehydrogenase. RBL-2H3 and RPMC cells (2 × 10^4^/well in 96-well plates) were pretreated with various concentrations of PD for 24 h and incubated with 1 mg/ml MTT at 37 °C. After 2 h, formazan crystals were dissolved with DMSO, and then absorbent intensity was detected using a spectrophotometer (Spectra MAX PLUS, Molecular Devices, Sunnyvale, CA, USA) at a wavelength of 570 nm. Absorbent intensity of formazan formed in untreated control cells was calculated as 100% viability.

### Passive cutaneous anaphylaxis (PCA) test and ear swelling response in mice

PCA reaction was generated by sensitizing skin with intradermal injection of 0.5 μg anti-DNP IgE in 50 μl phosphate-buffered saline. After 48 h, each mouse received intravenous injection of DNP-HSA (1 mg/mouse) and 4% Evans blue (1:1) mixture. PD was administered intraperitoneally at doses of 15, 30 and 40 mg/kg BW 1 h before challenge. Thirty minutes after challenge, the mice were euthanized, and both ears were collected to measure dye pigmentation and histology, followed by extraction of extravasated Evans blue dye by incubation of biopsies using 1 ml formamide at 55 °C for 24 h. Dye absorbance was measured at 620 nm using a spectrophotometer (Spectra MAX PLUS, Molecular Devices, Sunnyvale, CA, USA). The concentration of Evans blue was quantified by interpolation on a standard curve of dye concentrations in the range of 0.01 to 30 mg/ml. The ears were fixed with 4% formaldehyde and embedded in paraffin. Then, 5 μm sections were stained with toluidine blue to count the number of mast cells at five sites randomly selected for each sample under 100× magnification. The ear thickness was measured with a digital micrometer (Kawasaki, Japan) under mild anesthesia induced by intraperitoneal injection of 1:1 mixture (50 μl) of ketamine (1 mg/ml) and xylazine hydrochloride (23.32 mg/ml). Mice were kept in immobility state during the measurement.

### Histology and mast cell count

The ears were fixed with 4% formaldehyde and embedded in paraffin. Then 5 μm sections were stained with hematoxylin & eosin and toluidine blue (Sigma-Aldrich, St. Louis, MO, USA). The number of mast cells was counted in five randomly selected fields for each toluidine blue staining sample under 100× magnification. Three independent, blinded investigators counte the cells and interinvestigator variation was <5%.

### Anti-DNP IgE-Mediated Passive Systemic Anaphylaxis (PSA) in Mice

Mice were intravenously injected with 3 mg anti-DNP IgE. Twenty-four hours later, mice were challenged with intravenously administration of 500 mg of DNP-HSA or PBS. After 1.5 min, mice were sacrificed by cervical dislocation and blood was immediately collected by cardiac puncture. Serum was isolated from blood samples and tested for serum histamine concentration by the radioenzymatic method^53^. PD was The inhibition percentage of histamine release was calculated using the following formula: inhibition rate (%) = [1-(T-B)/(C-N)] ×100, Control (C): IgE (+), PD (−); Normal (N): IgE (−), PD (−); Test (T): IgE (+), PD(+); Blank (B): IgE (−), PD(+).administered intraperitoneally at doses of 15, 30 and 40 mg/kg BW 1 h before challenge.

### Histamine contents

Histamine contents in cultured cell supernatants were measured by radioenzymatic method. RBL-2H3 cells were preincubated with PD at 37 °C for 30 min prior to challenge with DNP-HAS (100 ng/mL). After centrifugation at 150 × g for 10 min at 4 °C, the supernatant was harvested for measurement of histamine contents. The inhibition percentage of histamine release was calculated as above.

### Measurement of 45Ca uptake

Calcium uptake of mast cells was measured following previously published procedures^11^. In brief, RPMCs were resuspended in HEPES-Tyrode buffer containing ^45^Ca (1.5 mCi/mL; 1 Ci = 3.7 × 1010 becquerels; Perkin-Elmer Life Sciences, Waltham, MA, USA) at 4 °C for 10 min. Mast cell suspensions were sensitized with 10 μg/ml anti-DNP IgE for 6 h and preincubated with prewarmed buffer containing various concentrations PD. The reaction was allowed to proceed for 30 min at 37 °C prior to challenge with DNP-HAS (100 ng/ml) and terminated by addition of 1 mM lanthanum chloride. The samples were centrifuged 3 times at 150 × g for 10 min, and then the mast cells were lysed with 10% Triton X-100 and vigorous shaking. Radioactivity was determined by a scintillation β-counter (Liquid Scintillation Analyzer, Canberra Industries, Inc., Meriden, CT, USA).

### RNA extraction and PCR

RBL-2H3 cells (5 × 10^5^/well in 6-well plates) were sensitized with anti-DNP IgE (50 ng/ml). After incubation overnight, the cells were pretreated with or without PD at 15, 30 and 40 mg/kg for 2 h and challenged with DNP-HSA (100 ng/ml) for 1 h. Then, the treated cells were harvested and total RNA was isolated using TRIzol extraction kit (Thermo Fisher Scientific, Waltham, MA, USA) according to the manufacturer’s instructions. cDNA was synthesized from 2 μg of total RNA using the PrimeScript RT-PCR kit (Takara, Dalian, China). RT-PCR was used to analyze the expression of mRNA for TNF-α, IL-4, IL-1β, IL-8, and β-actin (internal control). RT-PCR was performed using a Chromo 4 instrument (Bio-Rad, Hercules, CA, USA) and cycling conditions were as follows: 95 °C for 10 min; 40 cycles of 95 °C for 15 s, 60 °C for 1 min and 72 °C for 40 s. The amplified products were separated by electrophoresis on 2% agarose gel containing ethidium bromide, and documented using a molecular imager gel doc XR system (Bio-Rad, Hercules, CA, USA). All reactions were conducted in triplicate.

### Enzyme-linked immunosorbent assay (ELISA)

To measure the release of pro-inflammatory cytokines, ELISA was carried out using media from RBL-2H3 cells challenged with DNP-HSA and serum. ELISA was performed using an ELISA kit (Santa Cruz Biotechnology, Dallas, TX, USA) on a 96-well Nunc immune plate according to the manufacturer’s protocol. Before the detection of anti-DNP IgE, the immune plate was coated with 100 ng/mL DNP-HAS instead of a capture antibody. After terminating the reaction with a substrate, the absorbent intensity was detected using a spectrophotometer at a wavelength of 450 nm.

### Western blotting

Nuclear and cytosolic proteins were extracted as previously described^19^. Before protein extraction, RBL-2H3 cells (2 × 10^6^/well in 6-well plates) were sensitized with anti-DNP IgE (50 ng/ml). After incubation overnight, the cells were pretreated with or without PD for 1 h and challenged with DNP-HSA (100 ng/ml). Then, cell extracts were prepared by detergent lysis procedure as described previously^19^. Samples of protein (30 μg) were electrophoresed using 8–12% SDS-PAGE gel at 120 V for 90 min and transferred to polyvinylidene difluoride membranes (Amersham Pharmacia Biotech, Piscataway, NJ, USA). Immunodetection was carried out using a chemiluminescent substrate. The antibodies of IKK, phospho-IKK, NF-κB p65, phospho-NF-κB p65, PARP, IκBα, phospho-IκBα, and β-actin were purchased from Santa Cruz Biotechnology (Dallas, TX, USA). The antibodies of Syk, phospho-Syk, Lyn, phospho-Lyn, Gab2, phospho-Gab2, Akt, phospho-Akt, p38 MAPK, phospho-p38 MAPK, ERK, phospho-ERK, Nrf-2, and HO-1 were purchased from Cell Signaling Technology (Danvers, MA, USA).

### Lyn and Syk kinases assay

Activity of Lyn and Fyn kinases *in vitro* was measured using the Kinase Enzyme System and ADP-GloTM Kinase assay (Promega, Madison, WI, USA) according to the manufacturer’s instructions. Briefly, in a 96-well plate (25 mL total reaction volume), 0.1 mg each of Lyn and Syk was incubated in Reaction Buffer A (8 mM Tris, pH 7.5, 4 mM MgCl2, 0.02 mg/mL bovine serum albumin) supplemented with 50 mM dithiothreitol (DTT) for 10 min. Incubated kinases were mixed with 1 mg of each substrate peptide and 50 mM ATP, followed by incubation for 15 min. The mixtures were incubated at room temperature for 40 °C and 30 min after addition of ADP-GloTM Reagent and Kinase Detection Reagent, respectively. Luminescence was detected using a SpectraMax L apparatus (Molecular Devices) at a wavelength of 470 nm.

### Statistical analysis

All results were analyzed using SPSS 17.0 (IBM, Armonk, NY, USA). The data were presented as means ± standard error (SE) of independent experiments. Treatment effects were analyzed using a one-way analysis of variance followed by Duncan’s multiple range tests. A value of *P* < 0.05 was used to indicate statistically significant difference.

## Electronic supplementary material

supplementary information
