# Supplementary material for: Polydatin inhibits mast cell-mediated allergic inflammation by targeting PI3K/Akt, MAPK, NF-κB and Nrf2/HO-1 pathways
Source: Sci Rep. 2017 Sep 19;7:11895. doi: 10.1038/s41598-017-12252-3 (PMC5605538; doi:10.1038/s41598-017-12252-3)
Supplement: Supplementary file 1 — supplementary information [file 41598_2017_12252_MOESM1_ESM.pdf]

Polydatin inhibits mast cell-mediated allergic inflammation by targeting PI3K/Akt, MAPK, NF-κB and Nrf2/HO-1 pathways

Jing Ye[1,#], Hongmei Piao[2,#], Jingzhi Jiang[1], Guangyu Jin[2], Mingyu Zheng[3], Jinshi Yang[3], Xiang Jin[3], Tianyi Sun[3],Yun Ho Choi[4], Liangchang Li[1,*], Guanghai Yan[1,*]

[1]Department of Anatomy and Histology and Embryology, Yanbian University Medical College, Yanji 133002, P.R. China;

[2]Department of Respiratory Medicine, Yanbian University Hospital, Yanji, P.R. China;

[3]College of Pharmacy, Yanbian University, Yanji 133002, P.R. China;

[4]Department of Anatomy, Medical School, Institute for Medical Sciences, Chonbuk National University, Jeonju, Jeonbuk 561-756, Republic of Korea

#These authors contributed equally to this work.

*Corresponding authors:

Guanghai Yan: Department of Anatomy, Histology and Embryology, Yanbian University Medical College, No. 977 Gongyuan Road, Yanji 133002, Jilin Province, P.R. China

Tel.: 86-433-2435137

Fax: 86-433-2435136

Email: ghyan@ybu.edu.cn & ghyan2015@sina.com

Liangchang Li: Department of Anatomy, Histology and Embryology, Yanbian University Medical College, No. 977 Gongyuan Road, Yanji 133002, Jilin Province, P.R. China

Tel.: 86-433-2435137

Fax: 86-433-2435136

Email: lcli@ybu.edu.cn

Figure 3A

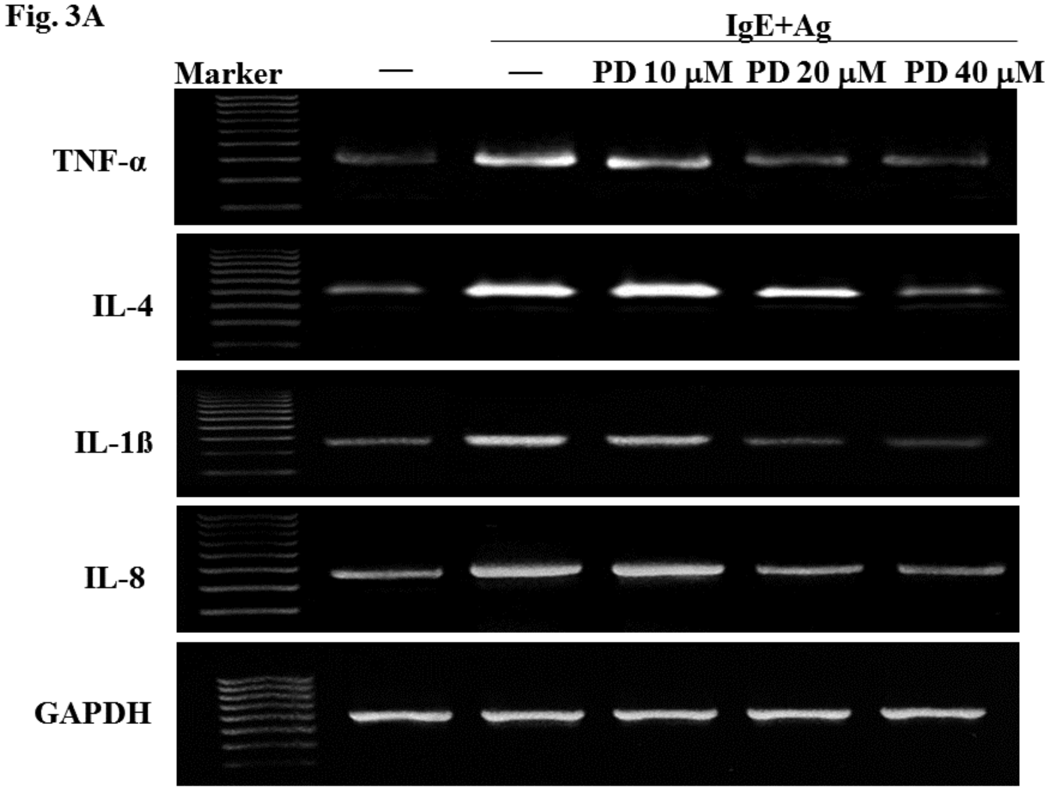
Fig. 3A
IgE+Ag
Marker
—
—
PD 10 μM
PD 20 μM
PD 40 μM
TNF-α
IL-4
IL-1ß
IL-8
GAPDH

Figure 4

Fig. 4

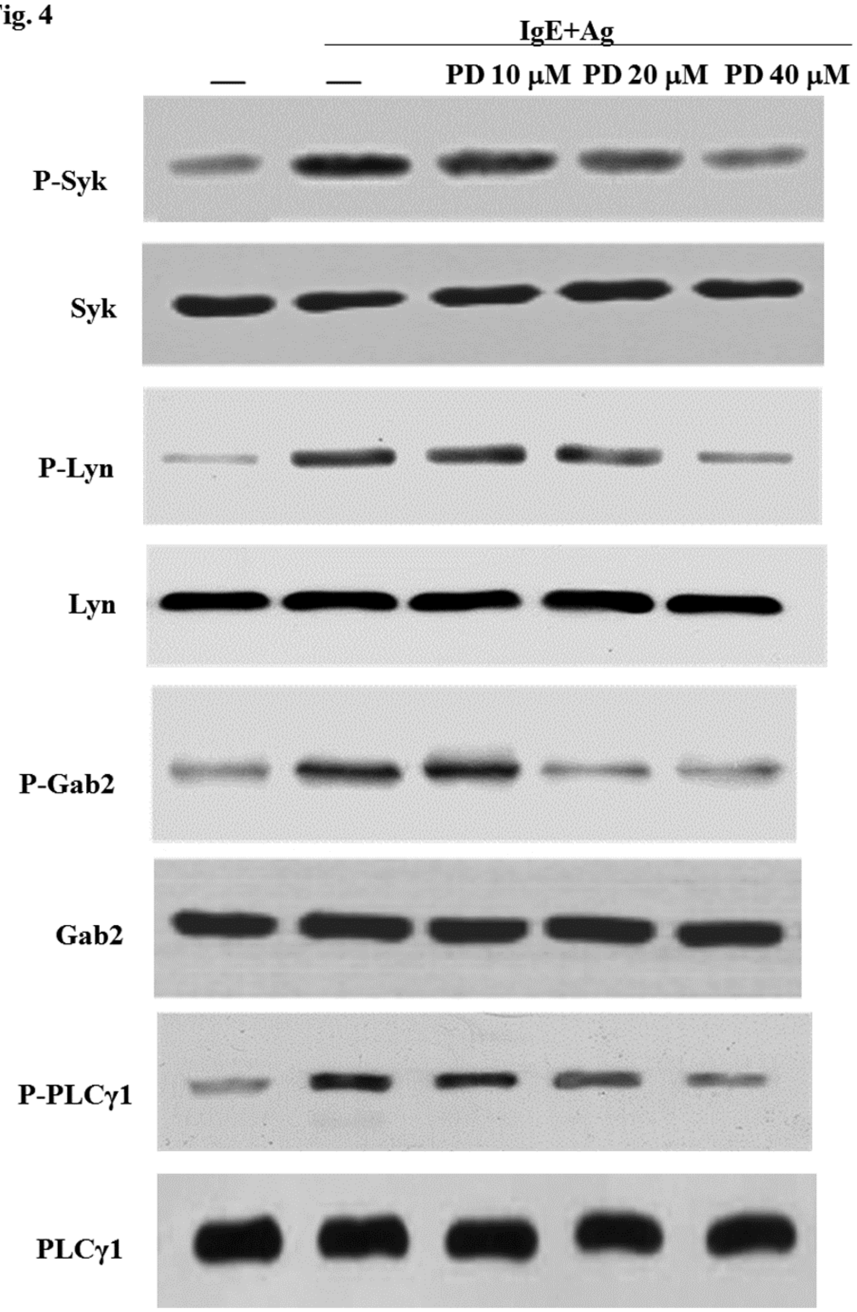

Figure 5

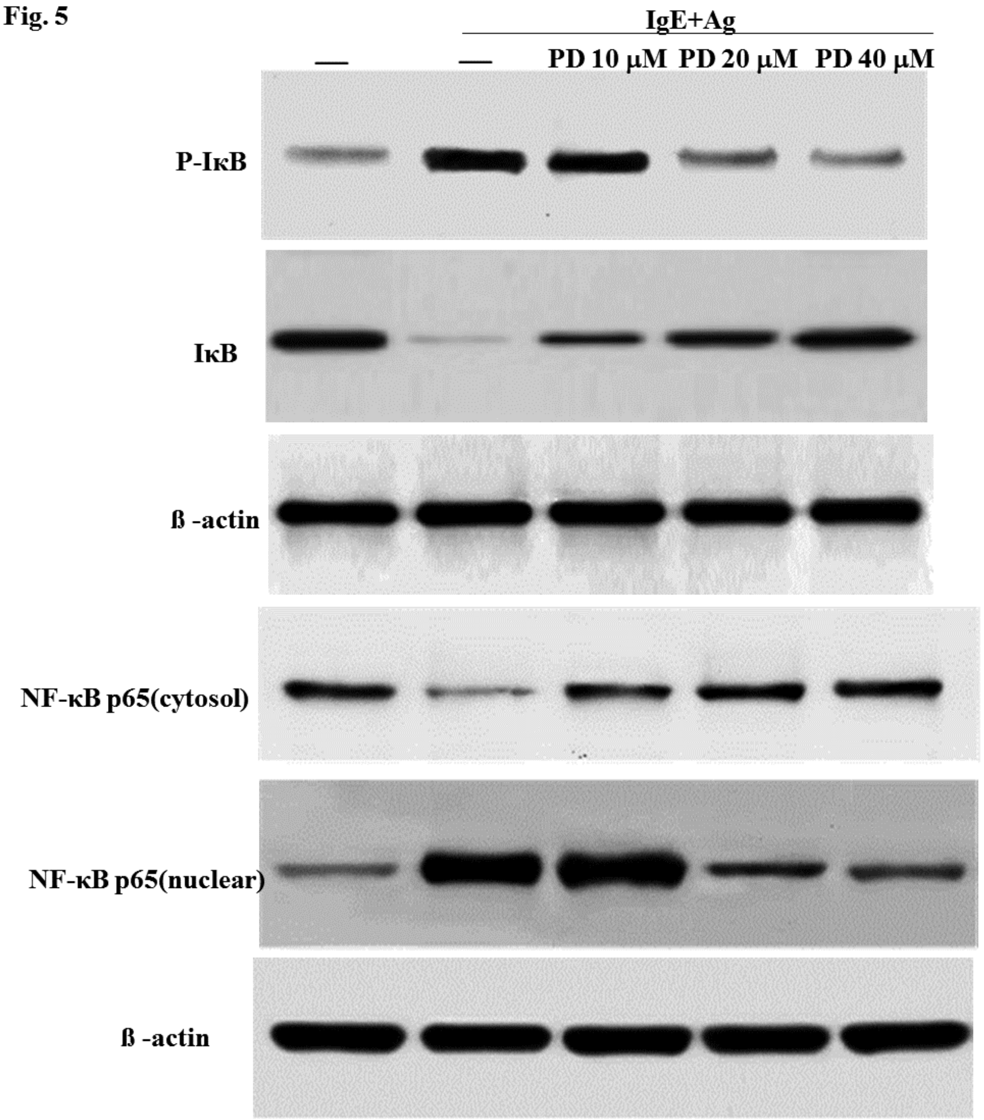
Fig. 5
IgE+Ag
—
—
PD 10 μM
PD 20 μM
PD 40 μM
P-IκB
IκB
ß -actin
NF-κB p65(cytosol)
NF-κB p65(nuclear)
ß -actin

Figure 6

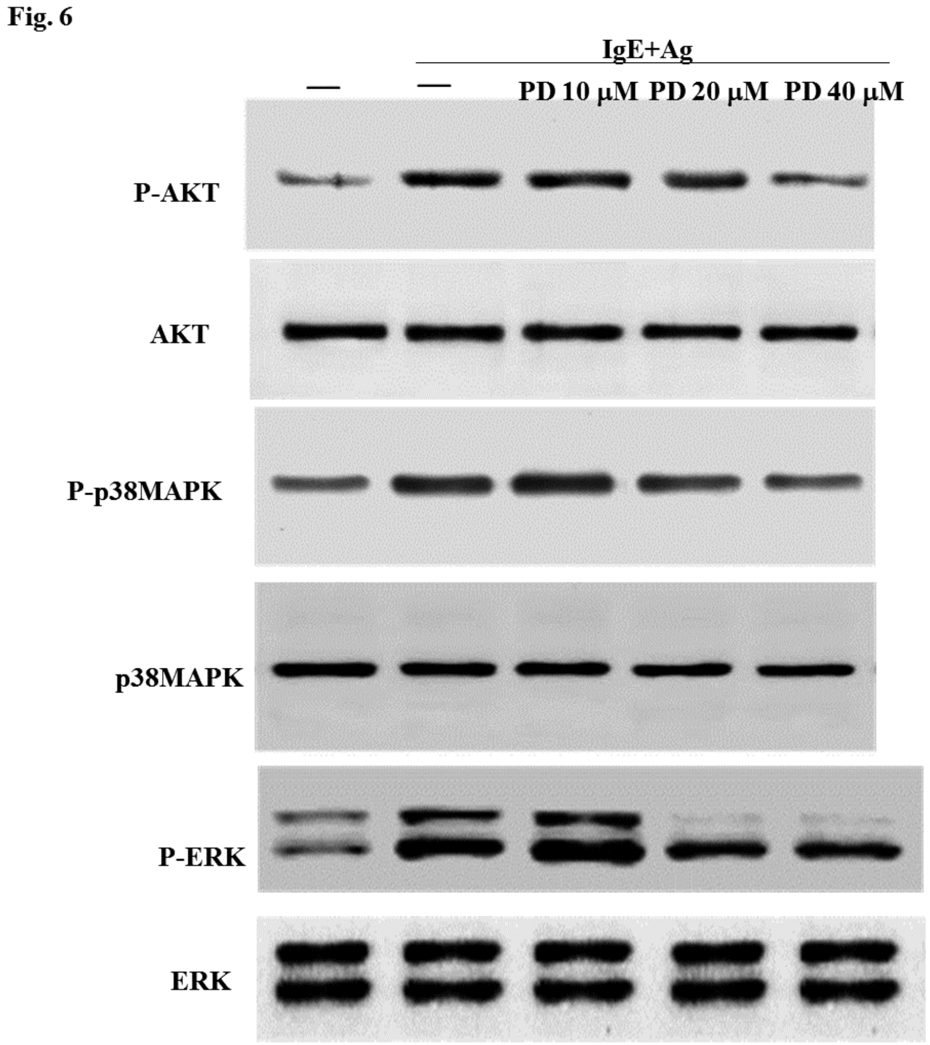
Fig. 6
IgE+Ag
—
—
PD 10 μM
PD 20 μM
PD 40 μM
P-AKT
AKT
P-p38MAPK
p38MAPK
P-ERK
ERK

Figure 7

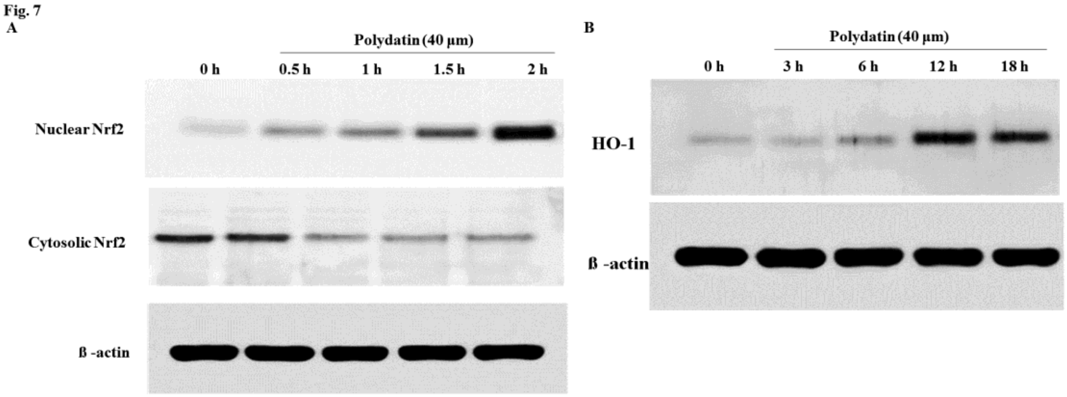
Fig. 7
A
Polydatin (40 μm)
0 h
0.5 h
1 h
1.5 h
2 h
Nuclear Nrf2
Cytosolic Nrf2
ß -actin
B
Polydatin (40 μm)
0 h
3 h
6 h
12 h
18 h
HO-1
ß -actin
